# Supplementary material for: Heavy Tau Burden with Subtle Amyloid β Accumulation in the Cerebral Cortex and Cerebellum in a Case of Familial Alzheimer’s Disease with APP Osaka Mutation
Source: Int J Mol Sci. 2020 Jun 22;21(12):4443. doi: 10.3390/ijms21124443 (PMC7352205; doi:10.3390/ijms21124443)
Supplement: Supplementary file 1 [file ijms-21-04443-s001.pdf]

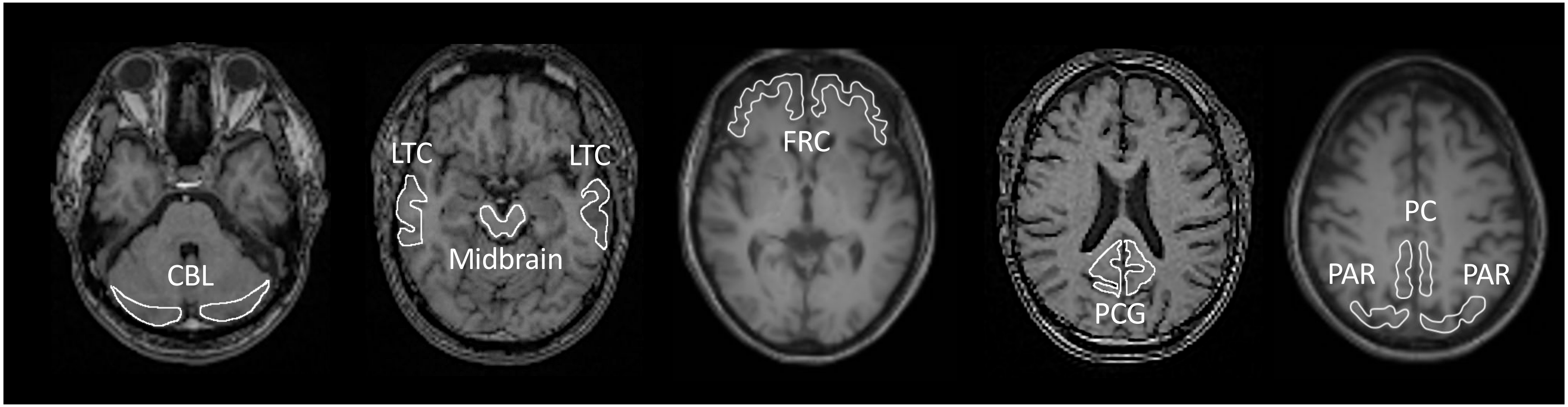

Supplementary Figure 1. Region of interest (ROI).
Each ROI was manually delineated on standard MRIs to which individual PET data was standardized after acquisition of individual MRIs. CBL: cerebellum, LTC: lateral temporal cortex, FRC: frontal cortex, PCG: posterior cingulate gyrus, PC: precuneus, PAR: parietal cortex.
